# Structural Insights Into Galectin‐3 Recognition of a Selenoglycomimetic

**DOI:** 10.1002/cmdc.202401005

**Published:** 2025-03-06

**Authors:** Maria Pia Lenza, Cristina Di Carluccio, Ferran Nieto‐Fabregat, Luciano Pirone, Rita Russo, Sonia Di Gaetano, Domenica Capasso, Alessia Stornaiuolo, Alfonso Iadonisi, Michele Saviano, Roberta Marchetti, Emilia Pedone, Alba Silipo

**Affiliations:** ^1^ Department of Chemical Sciences and Task Force for Microbiome Studies University of Naples Federico II Via Cinthia 4 80126 Naples Italy; ^2^ Department of Pharmacy University of Naples Federico II Via Domenico Montesano, 49 80131 Napoli Italy; ^3^ Institute of Biostructures and Bioimaging National Research Council (CNR) Via P. Castellino 111 80131 Naples Italy; ^4^ Department of Environmental Biological and Pharmaceutical Sciences and Technologies University of Campania “Luigi Vanvitelli” Via Vivaldi, 43 Caserta Italy; ^5^ Department of Physics “Ettore Pancini” University of Naples Federico II Via Cinthia 4 80126 Naples Italy; ^6^ Institute of Crystallography National Research Council (CNR) 81100 Caserta Italy; ^7^ CEINGE Biotecnologie Avanzate Franco Salvatore Via Gaetano Salvatore, 486 80131 Napoli NA

**Keywords:** Galectin-3, Selenoglycoside inhibitor, NMR, Carbohydrate recognition, Molecular Dynamics

## Abstract

Chimera‐type galectin‐3 (Gal‐3) is a β‐galactoside‐binding protein containing a single conserved carbohydrate‐recognition domain, crucial in fibrosis and carcinogenesis. Selenium‐based Gal‐3 inhibitors have emerged as promising therapeutic agents, particularly for treating neoplastic diseases. Among them, a seleno‐digalactoside (SeDG) substituted with a benzyl group at position 3 of both saccharide residues (benzyl 3,3′‐seleno‐digalactoside, SeDG‐Bn), attracted considerable attention for its selectivity and potent inhibitory efficacy against Gal‐3. NMR spectroscopy and molecular dynamics simulations were combined to investigate the binding of SeDG‐Bn to Gal‐3 at the molecular level. This approach revealed the recognized epitope, the binding mode within Gal‐3 binding pocket and enabled the generation of a 3D model of the complex. Our findings show that the presence of a single benzyl group establishes hydrophobic contacts with amino acids in Gal‐3 β‐sheets S2 and S3, crucially enhancing the binding affinity compared to unmodified SeDG. The digalactose backbone orientation in Gal‐3 binding site is partially modified by the benzyl group with respect to complexes with lactosamine and SeDG. These results provide valuable insights into the design of more potent and selective inhibitors for Gal‐3, potentially contributing to new therapeutic strategies for conditions such as cancer and fibrosis.

## Graphical abstract

Galectin‐3, a β‐galactoside‐binding protein, plays a pivotal role in fibrosis and carcinogenesis. We explore the molecular recognition of a seleno‐digalactoside, modified with a benzyl group (SeDG‐Bn), as a potent Gal‐3 inhibitor. Using nuclear magnetic resonance and molecular dynamics simulations, we present a 3D model of the SeDG‐Bn/Gal‐3 complex, highlighting enhanced binding affinity.

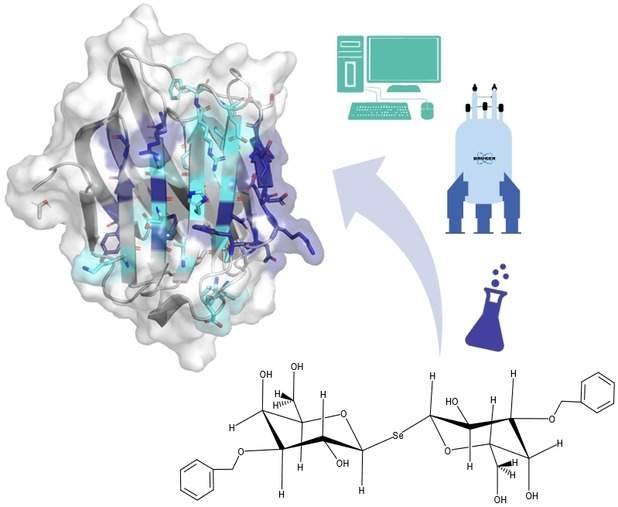

## Introduction

Galectins, a family of carbohydrate‐binding proteins, play a pivotal role in a myriad of physiological and pathological processes. These proteins, with their approximately 130 amino acids carbohydrate recognition domains (CRDs), exhibit a specific affinity for galactosides.^[1]^ Galectins are found in various cell types, including epithelial and endothelial cells from the skin, lung, gut, immune cells and fibroblasts and they are involved in several intracellular and extracellular processes.^[2]^

**Scheme 1:**
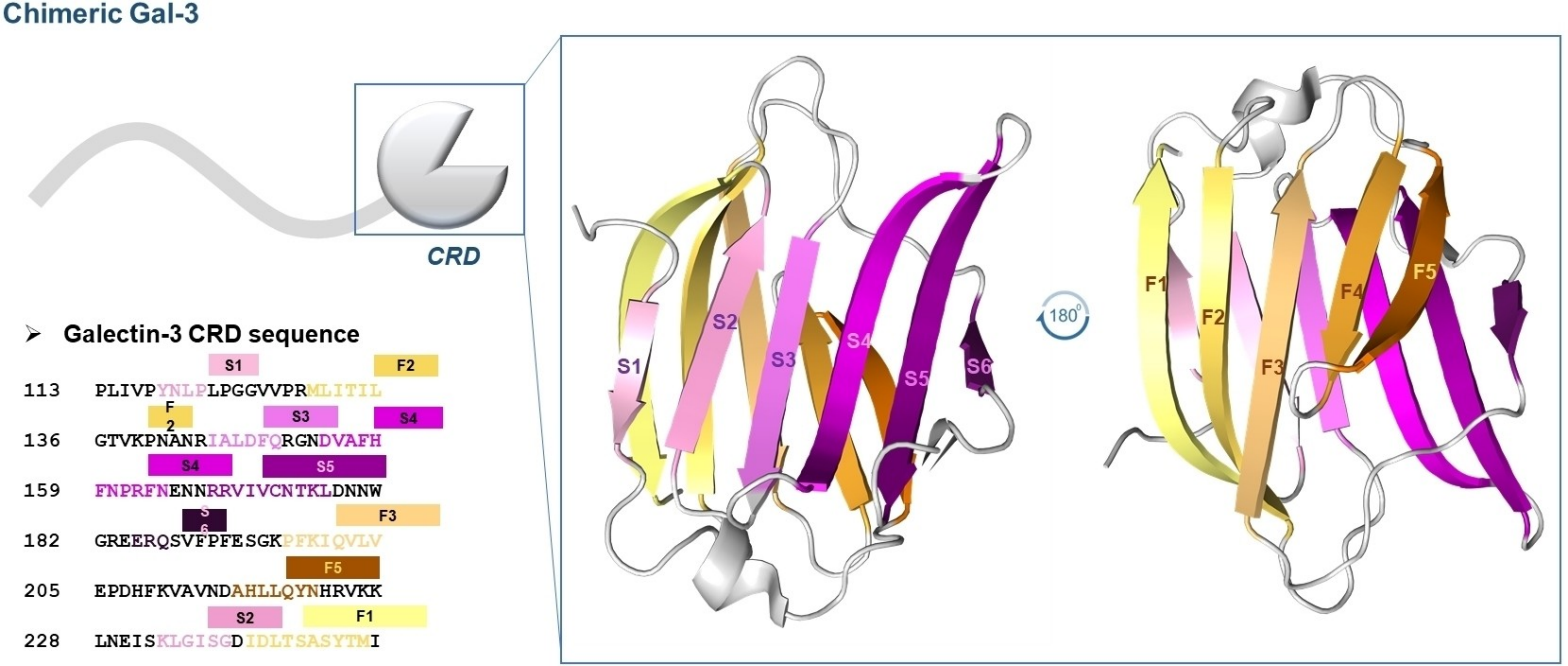
Schematic representation of chimeric Gal‐3. The amino acid sequence of Gal‐3‐and different views of the 3D structure of the protein (from PDB 3zsj) are shown. In the crystal structure of Gal‐3‐CRD, corresponding to the domain expressed and studied in this work, the β‐strands F1–F5 and S1–S5 are highlighted in shades of orange and pink, respectively.

Galectin‐3 (Gal‐3) is characterized by a single CRD to which a proline‐ and glycine‐rich N‐terminal domain is attached, and this additional domain gives Gal‐3 the ability to form complex oligomers (Scheme 1).[^3^, ^4^, ^5^]

Gal‐3 stands out for its broad impact across pathological and physiological processes.[^6^, ^7^, ^8^] Located within both the nucleus and cytoplasm and secreted into the extracellular environment, Gal‐3 modulates crucial cellular interactions, spanning from immune response modulation to cell adhesion, migration, and angiogenesis regulation. This wide range of action makes Gal‐3 a key player in the processes of fibrosis and carcinogenesis, where its expression is often up‐regulated and correlated with poor prognosis in several cancer types.[^9^, ^10^, ^11^, ^12^]

Gal‐3 inhibitors have emerged as a promising class of therapeutic agents, particularly for treating neoplastic diseases.[^13^, ^14^, ^15^, ^16^] Given its pivotal role in orchestrating pathways that promote fibrosis and tumorigenesis, there has been a concerted effort to identify compounds capable of precisely targeting Gal‐3 activity. Notably, derivatives of thio‐galactosides (TDG) have been explored for designing promising anti‐galectin agents.[^17^, ^18^, ^19^] In particular, TD139,[^20^, ^21^, ^22^, ^23^] a 3,3′‐deoxy‐3,3′‐bis‐(4‐[m‐fluorophenyl]‐1H‐1,2,3‐triazol‐1‐yl)‐thio‐digalactoside, has emerged as a small‐molecule glycomimetic inhibitor with high selectivity for the carbohydrate recognition domain (CRD) of Gal‐3. Modifications at position C3 of TDG introduce additional favorable protein‐ligand interactions, resulting in a 1000‐fold increase in affinity. The resulting fluorophilic microenvironment enables multiple polar interactions between the terminal fluorine of TD139 and protein backbone atoms, as reported by Hsieh et al.^[24]^ Several studies have demonstrated that TD139 exhibits anti‐inflammatory effects due to its ability to reduce Galectin‐3 expression.[^25^, ^26^] In preclinical and clinical studies, TD139 has shown remarkable efficacy in treating pulmonary fibrosis and altering the tumour microenvironment by reducing tumor cell proliferation, invasion, and metastasis.[^6^, ^27^] Nevertheless, its development was hindered by the emergence of adverse effects in clinical trial,^[28]^ emphasizing the need to investigate alternative approaches to galectin‐3 inhibition.

In this regards, beyond traditional inhibitors, there is growing interest in the role of selenium‐containing compounds in modulating Gal‐3. André group pioneered the substitution of the glycosidic oxygen with selenium, a strategy that, along with disulfide linkages, yields hydrolysis‐resistant glycosides.^[29]^ Selenoglycosides are highly sought after due to their increasing biomedical potential and their practical applications as probes for X‐ray and Se NMR techniques.^[30]^ Selenium has been studied for its ability to modulate inflammatory pathways,[^31^, ^32^] which could theoretically impact expression or activity of galectins. Some studies suggest that selenium supplementation can reduce inflammatory markers in conditions like arthritis and cardiovascular diseases, potentially influencing galectin‐related pathways.^[33]^ Organoselenides have consistently attracted scientific interest for their anticancer and anti‐inflammatory properties, owing to the unique dual nature of selenium. Unlike sulphur, the lighter chalcogen, selenium exhibits both antioxidant and pro‐oxidant characteristics. The development of a selenoglycosidation method using inexpensive elemental selenium and reactive glycosyl iodides^[30]^ has mitigated the higher cost traditionally associated with selenoglycoside synthesis compared to sulphur‐containing analogues, which stemmed from the use of expensive commercial selenating agents.

A notable example of a selenoglycomimetic is represented by the seleno‐digalactoside, (di(β‐D‐galactopyranosyl)selenide) from here on named as SeDG. This compound has been studied for its interaction with human galectins, particularly Gal‐1 and Gal‐3.^[34]^ SeDG, featuring a unique selenoglycoside linkage between two galactose units, unlike the typical oxygen‐centered bonds in other glycoside inhibitors, has been evaluated for its ability to bind and inhibit galectin activity using various biological and biophysical methods. These findings revealed that SeDG can strongly interact with Gal‐3, offering potential as a therapeutic agent in treating conditions associated with Gal‐3 overexpression, such as fibrosis and cancer. Additionally, in a recent study, SeDG was chemically modified by adding a benzyl group to position 3 of the saccharide residues, to give a benzyl 3,3′‐seleno‐digalactoside (benzyl 3,3′‐di(β‐D‐galactopyranosyl)selenide) from here on named as SeDG‐Bn.^[35]^ It has been proposed that this derivatization at C3 improves affinity for galectins through favorable interactions with charged residues.^[36]^ While modifications at C4 and C6 are possible, the hydrogen bonding network between the β‐galactoside scaffold and galectins are essential for both stabilizing β‐galactoside binding and ensuring substrate recognition. Consequently, we targeted position 3 for modification, adding an aromatic benzyl group at this specific location on both sugar moieties. This choice was supported by previous molecular docking studies, which indicated that benzylation at this position would enhance protein binding.^[35]^ In particular, the presence of the phenyl rings in SeDG‐Bn allows the formation of π‐cation interactions with the side chains of R144 and R186. Finally, this modification increased binding affinity and proved notable antiproliferative, anti‐migratory, and antiangiogenic properties, rendering SeDG‐Bn a promising anti‐cancer agent.^[35]^ Here, we investigated the details of this molecular interaction analyzing the binding between SeDG‐Bn (Scheme S1) and Gal‐3 by using nuclear magnetic resonance (NMR), from both protein and ligand perspective, combined with molecular dynamics simulation. This approach allowed us to explore in detail the 3D complex, evaluating the recognition and binding process and therefore providing a deeper understanding of the molecular mechanisms underlying its inhibitory activity.

## Results and Discussion

### Ligand‐Based NMR Experiments to define SeDG‐Bn Epitope Mapping

**Figure 1:**
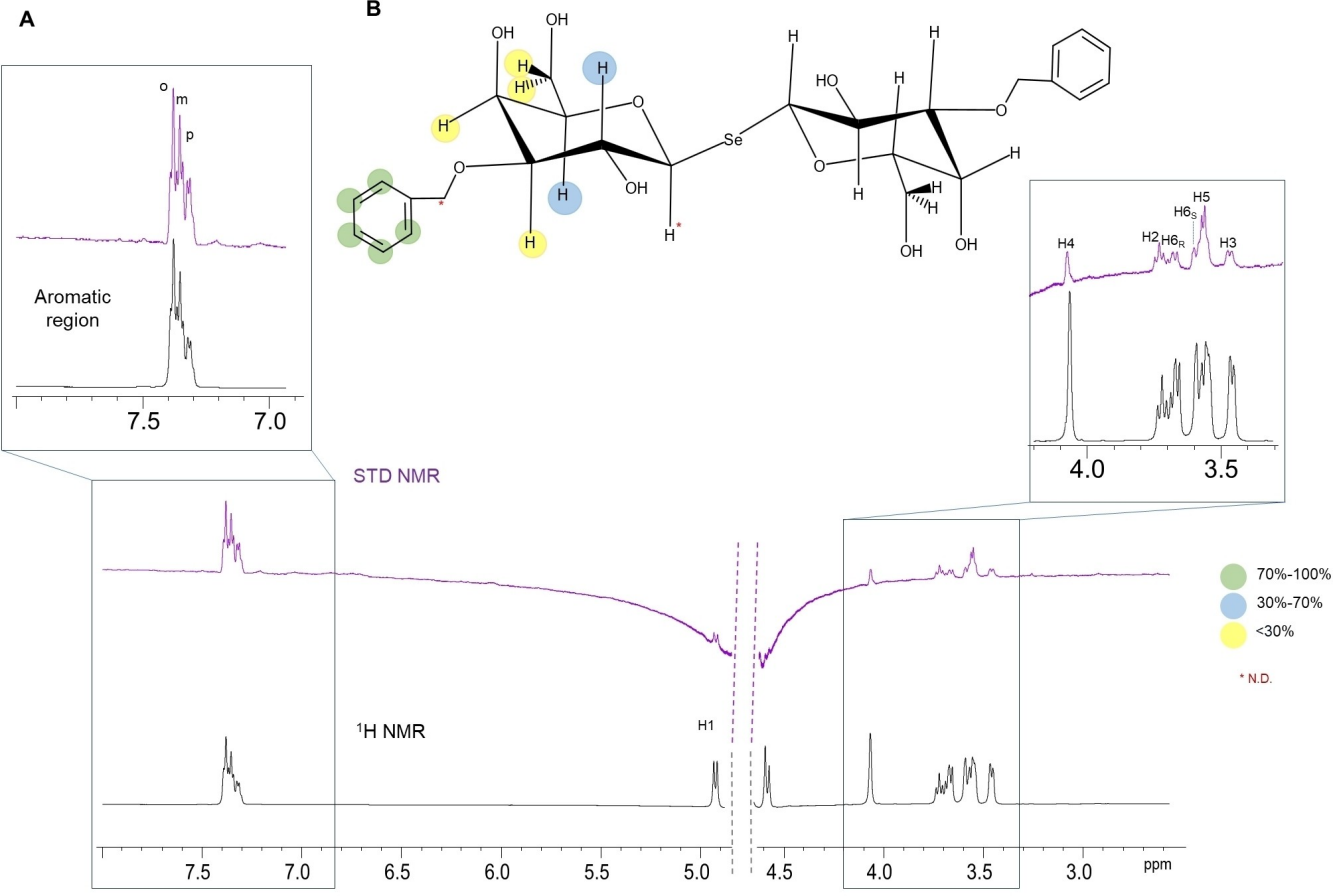
STD NMR analysis of SeDG‐Bn with Gal‐3. (A) STD NMR spectrum (purple) and the off‐resonance spectrum (black) of the 1 : 50 mixture of Gal‐3 : SeDG‐Bn. (B) The epitope mapping of SeDG‐Bn was depicted considering the combination of TD NMR intensities and computational analysis.

The interaction between Gal‐3 and SeDG‐Bn was confirmed using ligand‐based NMR experiments. Binding of SeDG‐Bn to Gal‐3 was supported by a decrease in ligand proton T2 relaxation times in CPMG experiments (Figure S1and confirmed by Saturation Transfer Difference (STD) NMR which further mapped the specific binding epitope (Figure 1). According to the observed signal enhancements in the STD, relative to the reference spectrum, the aromatic ring received over 70 % of the saturation from the protein, exhibiting stronger STD responses than the sugar moiety,, with the para‐positioned proton showing the highest STD effect. Protons at positions 2 and 5 of galactose exhibited moderate STD effects, at approximately 30 % and 46 %, respectively; those at positions 3, 4, and 6 showed considerably weaker STD effects, below 30 %. Although low STD signals were detected for the anomeric proton and methylene groups of the benzyl moiety, they were not quantified due to their proximity to the water (HDO) signal.

Therefore, STD NMR analysis elucidated the SeDG‐Bn binding epitope, demonstrating preferential binding of Gal‐3 to the aromatic protons, with some contribution from galactose protons. Nevertheless, the symmetry of SeDG‐Bn prevented NMR from distinguishing signals between the two aromatic rings and the two galactose residues. The epitope shown in Figure 1, depicting two of the four Bn and Gal units bound to Gal‐3, and excluding the other two from recognition, was achieved by combining STD NMR experiments with computational methods, this latter further discussed below.

### Protein‐Based NMR Experiments to Identify Gal‐3 Binding Site Interacting with SeDG‐Bn

Information detailing the binding site of Gal‐3 involved in the interaction with SeDG‐Bn was obtained by the analysis of the chemical shift perturbation through 2D ^1^H‐^15^N HSQC NMR spectra on the ^15^N‐labelled CRD of Gal‐3, expressed and purified as detailed in the experimental section. The NH spin systems were identified following the protein assignment of Umemoto *et al*.^[37]^

**Figure 2:**
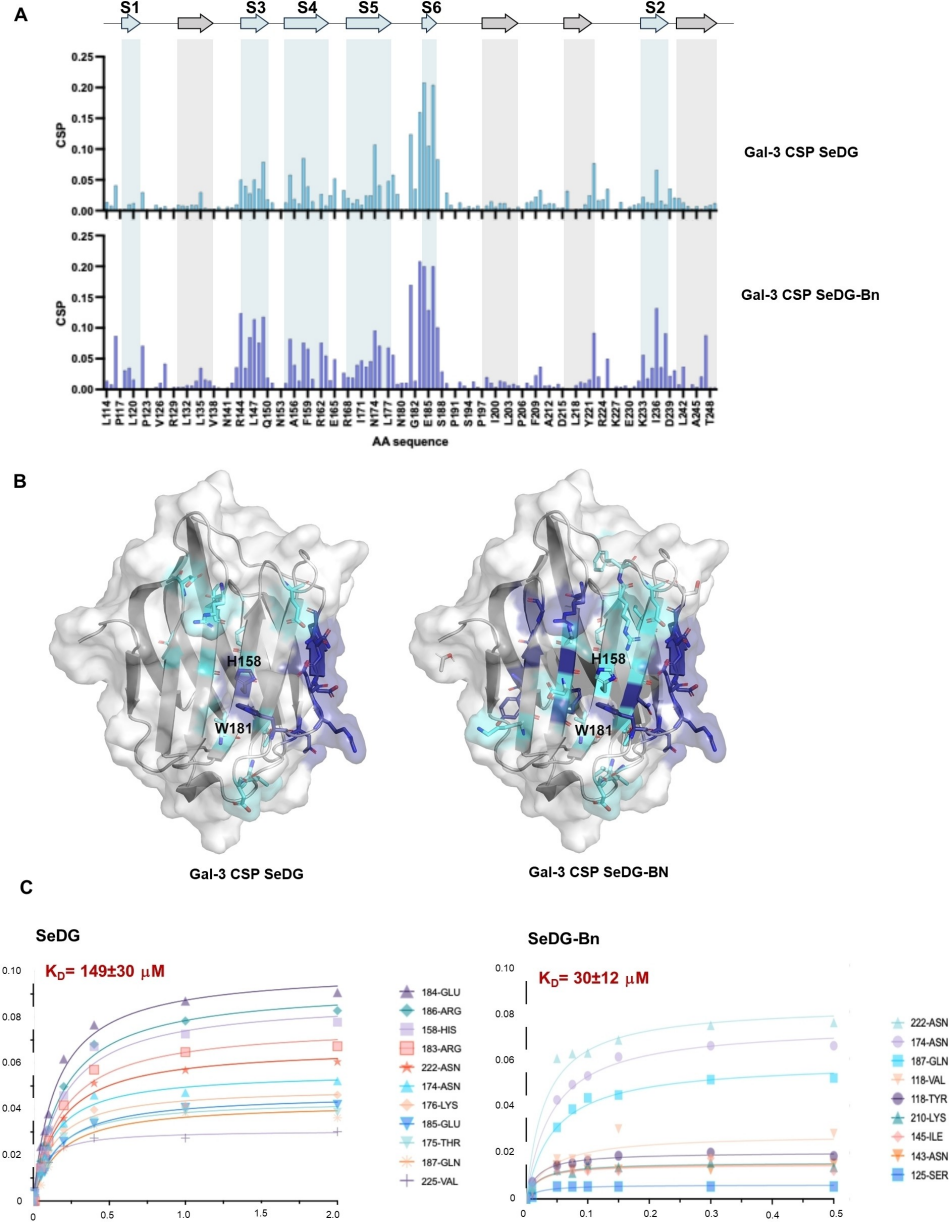
NMR Chemical shift perturbation. A) TROSY resonance maps showing the binding interactions between Gal3 and SeGD or SeDG‐Bn. Changes in Gal3 CSP observed for Gal3CRD in the presence of each ligand are plotted vs. the amino acid sequence of Gal3. The blue bars represent the CSP of SeDG, while the purple SeDG‐Bn. B) 3D views of Gal‐3 structure (PDB 3ZJS) colored according to the amino acids affected by stronger (Blue) and fewer (Cyan) CSP effects due to the presence of SeDG (left) and SeDG‐Bn (right). C) KD values determined by fitting the combined 1H/15N chemical shift changes (Δδ) of 15N‐labeled Gal3 observed in 2D 1H,15N‐TROSY spectra upon titration with A. SeDG and B. SeDG‐Bn.

Chemical shift perturbation (CSP) experiments were performed to monitor the binding of SeDG and SeDG‐Bn to [U−^15^N] Gal‐3. Each ligand was titrated into the protein, and changes in chemical shift were observed, primarily for amino acids at the binding interface and involved in the interaction. We could therefore compare the accommodation in the binding site of the two selenium‐containing compounds (Figure 2).^[38]^ As described, in the presence of SeDG (Figure 2A, upper part), specific CSPs were clearly observed in the β‐sheets S4–S5–S6, which constitute the canonical binding site.[^39^, ^40^, ^41^, ^42^] Among them, CSPs in His158, Arg162, Asn174, and Glu184, which establish hydrogen bonds with galactose, were observed. Additionally, the Gal unit formed CH‐π interactions with Trp181.

Analogies in the CSP map obtained by titration experiments with SeDG‐Bn (Figure 2A, lower part) compared to SeDG indicated that the accommodation mode of the two ligands was comparable. Nevertheless, key differences were evident, reflecting the impact of the benzyl substitution on ligand recognition. While SeDG primarily interacts with canonical binding site residues (His158, Arg162, Trp181), SeDG‐Bn perturbs additional residues in β‐sheets S2 and S3, such as Leu147 and Phe149, suggesting that the benzyl group establishes novel hydrophobic interactions.

Analysis of the CSPs also allowed to calculate the equilibrium dissociation constants (K_D_) of the hGal‐3 (CRD) in complex with SeDG and SeDG‐Bn using the residues that displayed the most significant chemical shift perturbations upon binding (see Figure 2C). The fitting of the binding curves resulted in a K_D_ value of 149±30 μM and 30±12 μM (Figure 2C), respectively, in agreement with previous literature,[^35^, ^38^] which reports that the introduction of the benzyl group at C3 of SeDG‐Bn leads to an increase in binding affinity compared to SeDG. Thus, these perturbations, alongside the higher binding affinity observed for SeDG‐Bn, underscore the role of the benzyl group in modulating both binding specificity and affinity. Furthermore, the protein saturation was achieved with significantly fewer equivalents of SeDG‐Bn (5 equivalents) compared to SeDG (20 equivalents), consistent with a stronger binding. In the NMR chemical shift timeframe, the exchange rate between the free and bound forms of Gal‐3 was slow in the presence of SeDG‐Bn, as indicated by decreases of some cross peak signal intensities. This behavior contrasted with that observed for SeDG, where a progressive shift of cross‐peak signals indicated a binding event in the fast‐exchange regime. These results collectively suggest that the benzyl group not only enhances affinity but also contributes to a tighter and more stable interaction within the Gal‐3 binding site.

Increased perturbation in amino acids within the β‐sheets S2 and S3 further highlighted the role of the benzyl group, which appeared to interact, in a hydrophobic region, with apolar amino acids, such as Leu147, Ile236, and Gly238, and hydrophobic amino acids, like Phe149 (Figure 2). In contrast, these amino acids located in S2 and S3 β‐sheets were not significantly perturbed when Gal‐3 interacted with SeDG, due to the absence of the aromatic moiety. Additionally, CSPs in charged amino acids, specifically Arg144, Asp148, and Lys233, displayed high perturbation levels in response to SeDG‐Bn. These findings suggest that that the benzyl group occupies a distinct hydrophobic region while maintaining solvent exposure of the Bn residue, since the CSPs of the amino acids belonging to S6 β‐sheet in the presence of SeDG‐Bn were comparable to those detected in the SeDG. Overall, the CSP data and their alignment with K_D_ values provide a comprehensive view of how the benzyl substitution impacts ligand recognition, shedding light on the intricacies of SeDG‐Bn : Gal‐3 binding dynamics.

### Computational Studies to Define the 3D Gal‐3/SeDG‐Bn Complex

**Figure 3:**
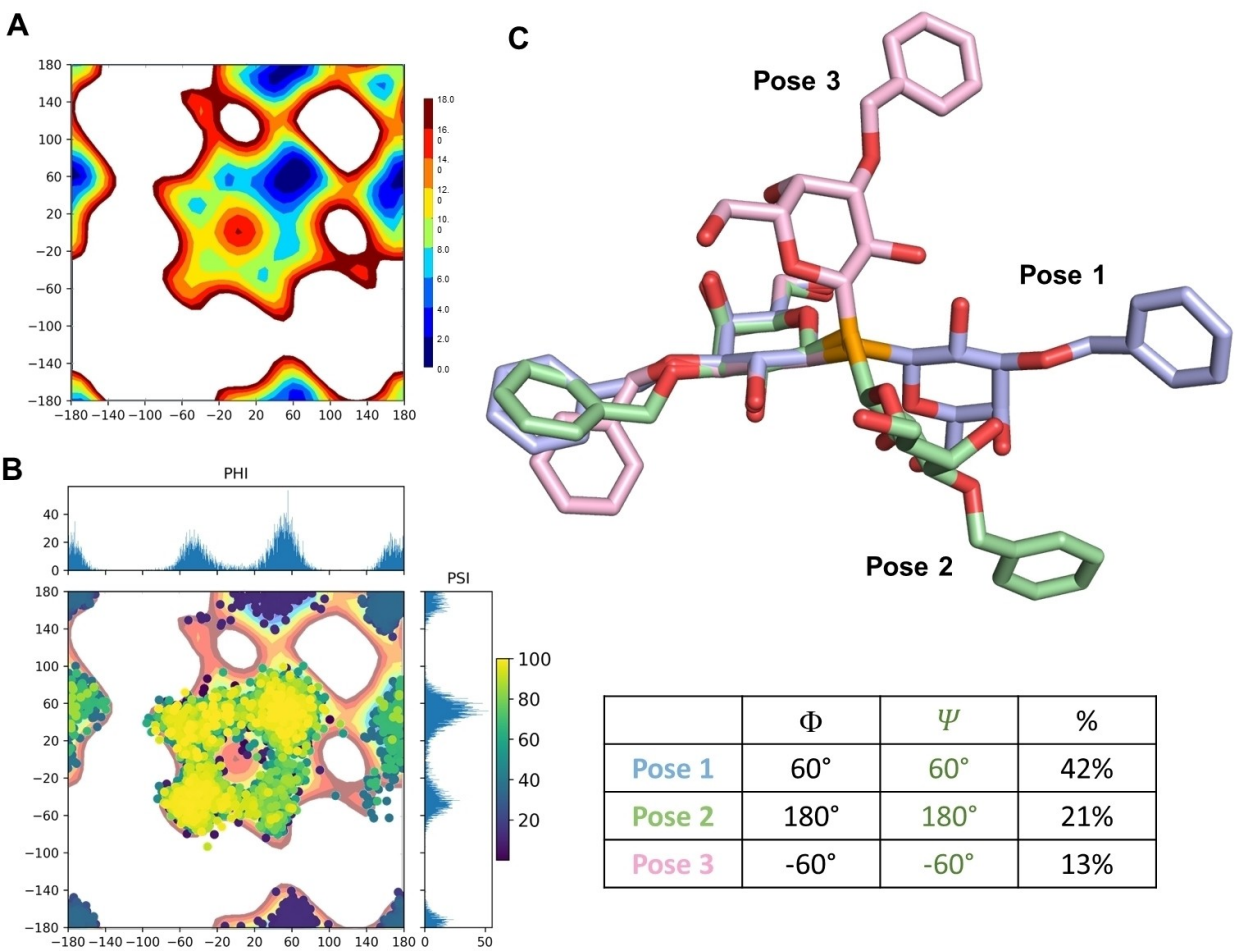
MM and MD studies of SEDG‐Bn conformations in the free state. A) Adiabatic energy maps, illustrating the glycosidic torsion angles of SeDG‐Bn obtained by molecular mechanics simulations. B) SeDG‐Bn free state dihedral angles analysis represented as scatter plots of the Phi torsion against Psi during the MD simulation with the relative histograms to represent the most populated energies. C) A three‐dimensional representative model coming from the 100 ns MD for SeDG‐Bn in free state.

Molecular dynamics (MD) simulations were performed to study the conformational characteristics of seleno‐glycosides, both in their free state and when bound to Gal‐3.^[43]^ Given the unique properties of selenium, and the lack of existing parameters for this atom, parameterization of the selenium glycoside was essential to perform the MD simulations with AMBER18.^[44]^ The SeDG ligands were indeed parametrized using Gaussian 09^[45]^ and VFFDT^[46]^ as previously reported.^[34]^ Thus, the disaccharide backbone constituting SeDG and SeDG‐Bn was constructed to evaluate energetically accessible conformational regions via molecular mechanics (MM) calculations using standard protocols. Adiabatic energy maps (Figure 3A), illustrating the φ/ψ glycosidic torsion angles, namely φ (H1−C1−Se−C1’)/ψ (C1−Se−C1’−H1’), confirmed that the global energy minimum aligns with the *exo‐anomeric* effect.

Docking calculations were performed between Gal‐3 and SeDG‐Bn using CB‐Dock2^[47]^ for cavity detection and blind docking, followed by targeted docking with AutoDock Vina.^[48]^ CB‐Dock2 analysis identified a primary cavity within the carbohydrate recognition domain, encompassing residues from β‐sheets S2 to S6. Consistent with protein‐based NMR results, docking calculations highlighted the presence of residues Trp181, Arg162, and Glu184 in ligand recognition. The most populated docking cluster also corresponded to the lowest energy configuration, emphasizing the reliability of this orientation as a starting structure. Indeed, the most favorable pose from this less energetic and most populated family revealed critical hydrophobic interactions between the benzyl group of SeDG‐Bn and residues Leu147 and Phe149, alongside canonical hydrogen bonds involving the galactose moiety and residues Arg162 and Glu184 (Figure S2). These interactions aligned well with experimental data, reinforcing the role of the benzyl group in enhancing ligand specificity and stability. Additionally, docking calculations were also performed for TD139 using the same procedure (CBDock and AutoDock Vina) to compare its binding mode with that of SeDG‐Bn. The results revealed only minor differences in the binding poses, likely attributable to ligand‐specific structural variations, but with no significant changes in the overall interaction pattern (Figure S2).

**Figure 4:**
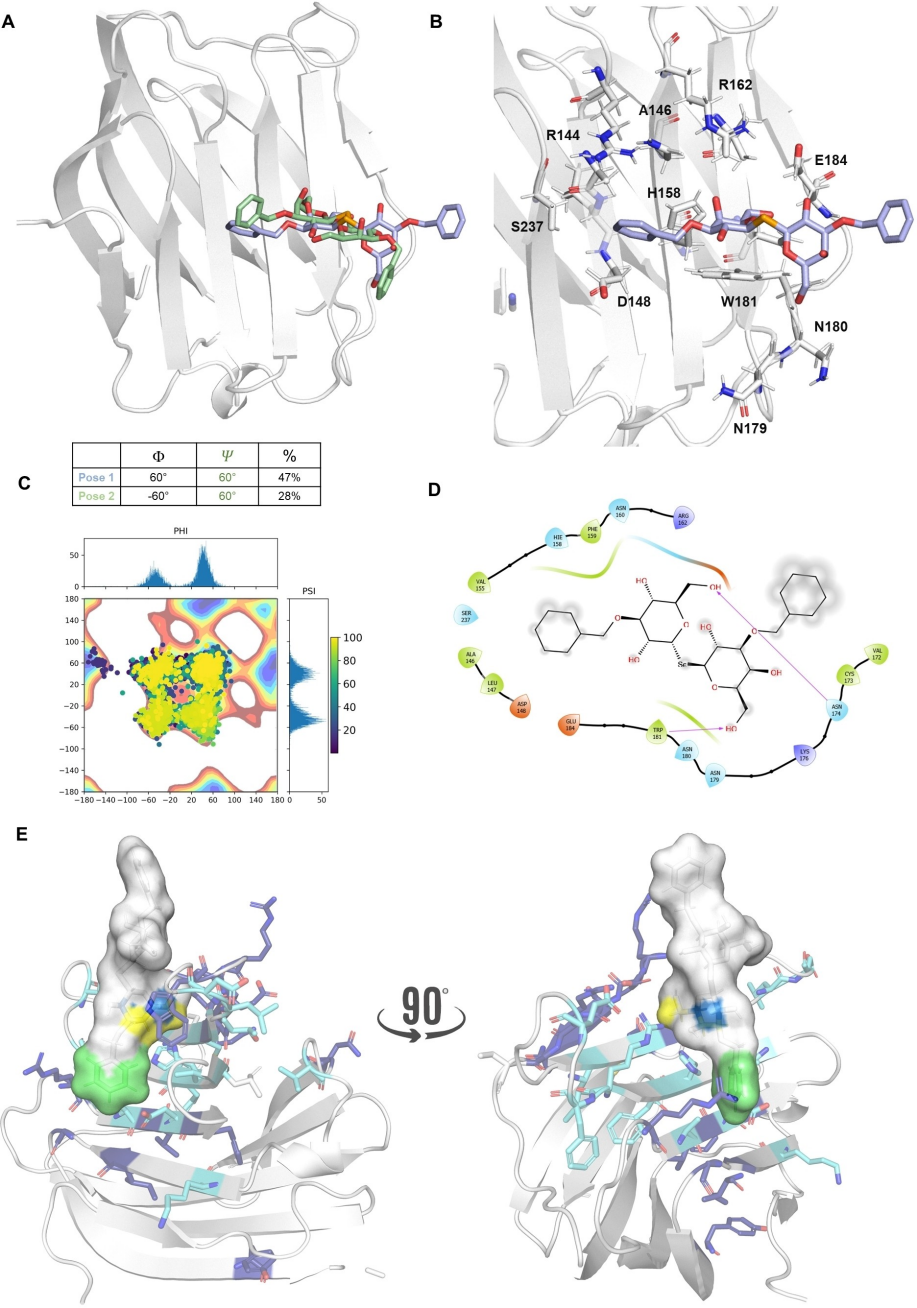
A three‐dimensional representative model coming from the 100 ns MD for the complex SeDG‐Bn and Gal‐3. A) the three representative conformations adopted by SeDG‐Bn in complex with Gal‐3 with the respective φ and ϕ angles values. B) A three‐dimensional representative model coming from the 100 ns MD for the complex SeDG‐Bn and Gal3. The amino acids involved in the interaction are shown as sticks. C) SeDG‐Bn bound state dihedral angles analysis represented as scatter plots of the Phi torsion against Psi during the MD simulation with the relative histograms to represent the most populated energies. D) Two‐dimensional schematic plot of the interactions between the Gal‐3 and SeDG‐bn. E) Gal‐3−SeDG‐Bn complex with Gal‐3 coloured as revealed by protein‐based NMR. SeDG‐Bn is coloured according to the STD edit code.

Therefore, this pose was selected for subsequent molecular dynamics (MD) simulations, performed with AMBER 18^[43]^ in explicit water, to further explore stability, dynamics and hydration of the Gal‐3/SeDG‐Bn complex. RMSD (Root Mean Square Deviation) calculations were performed on both the protein backbone and ligand residues, using the first frame as a reference, to provide evidence of complex stability. Moreover, the φ/ψ glycosidic torsion angles were monitored both in the free and the bound state. Regarding the free state, SeDG‐Bn exhibited a high degree of freedom, adopting extended (60°/60°), semi‐extended (−60°/‐60°), and closed (180°/180°) conformations (Figure 3B, Figure 3C). However, in the bound state, only the extended and semi‐extended conformations were permitted, suggesting that a conformer selection occurs upon binding (Figures 4). In all the most representative poses, SeDG‐Bn was similarly accommodated into the binding site of Gal‐3, with both Bn and Gal recognized by the protein, and Gal’ and Bn’ solvent exposed, due to the flexibility of the linkage. The galactose moieties of both Lac and SeDG were accommodated in the canonical Gal‐3 binding pocket as reflected in the X‐ray structure (PDB : 3ZSJ).^[41]^ These interactions are consistent with the known galactose binding mode, where the electropositive face of the galactose aligns with the indole moiety of the key residue Trp181, forming a stable CH‐π interaction.^[49]^ Specifically, C−H bonds at positions 3, 4, 5, and 6 of the galactose are positioned approximately 4 Å away from Trp181‐. Additionally, the internal galactose forms a hydrogen bond with His158, maintaining a typical binding conformation.

**Figure 5:**
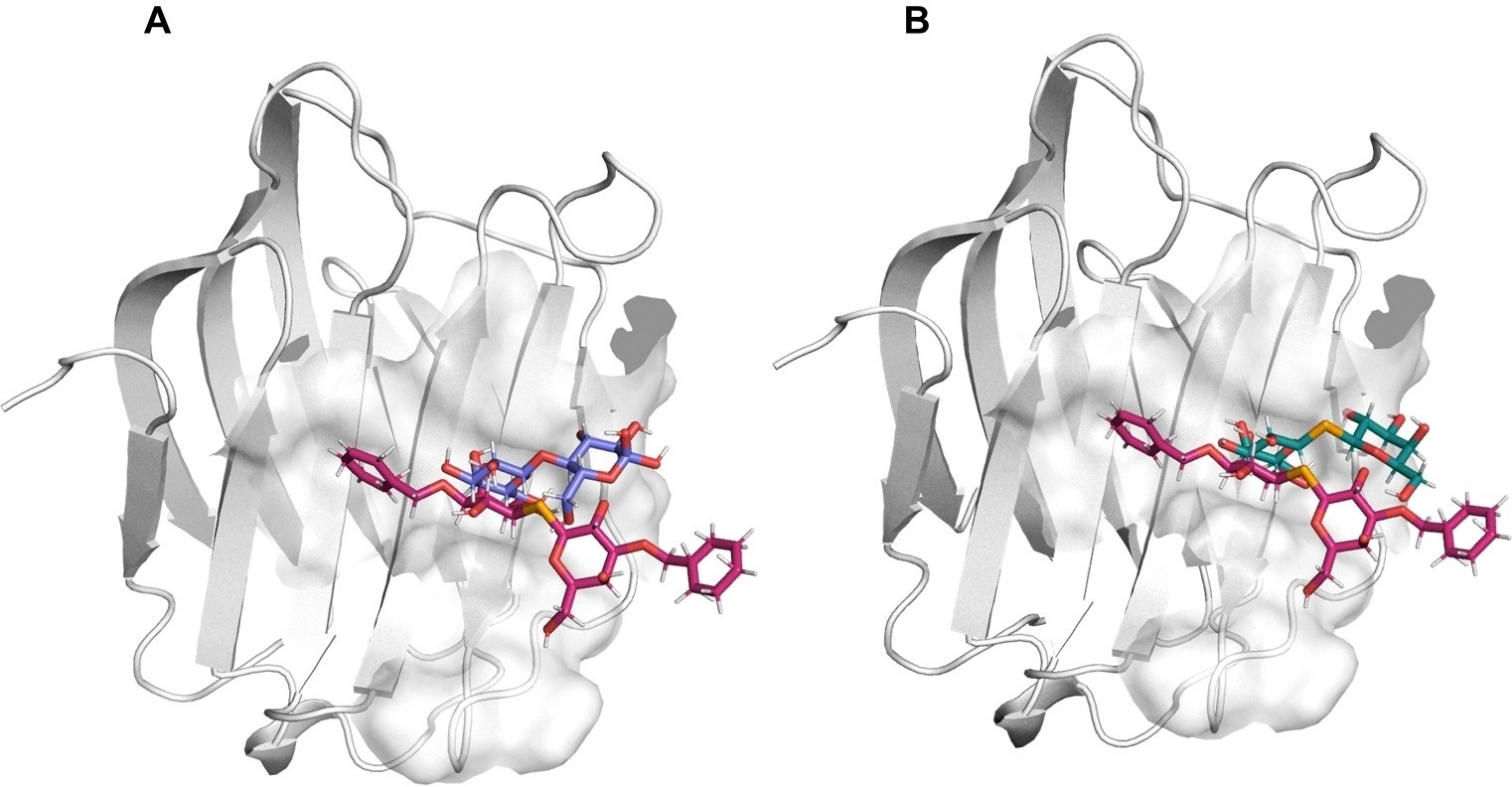
Superimposition of the complex between Gal‐3 and Lac/SeDG/SeDG‐Bn. A. Full view of the 3‐D complexes of Lac*N*Ac (purple, 3ZSJ) and SeDG‐Bn (pink) with Gal‐3 (obtained by MD simulation). B. Full view of the 3‐D complexes of SeDG (green) and SeDG‐Bn (pink) with Gal‐3 obtained by MD simulation.

In SeDG, this canonical binding mode is preserved, with the Gal’ moiety occupying the Gal‐3 binding site and forming hydrogen bonds with Glu184 and Arg186 via its protons at positions 2 and 3. However, the introduction of the benzyl group in SeDG‐Bn modulates this interaction by inducing a shift in the orientation of the glycosidic linkage (Figure 5). Computational studies reveal that the presence of the benzyl group alters the ψ angle from 0° to 60° around the seleno‐glycosidic bond, moving the Gal’ residue away from the Gal‐3 binding site, no longer forming the hydrogen bonds observed for SeDG.

Despite these changes, the most stable binding pose of the Gal‐3/SeDG‐Bn complex – identified through cluster analysis of molecular dynamics simulations (Figure 4, Pose 1) – retains the CH‐π interaction between Trp181 and the galactose. Nevertheless, the presence of the benzyl group partially displaces Gal and Gal’ compared to the X‐ray structure.

Beyond the CH‐π interactions between Trp181 and Gal ring as well as the van der Waals contacts of its aliphatic protons with the indole ring, other contacts were predicted (Figure 4). Stable polar interactions were observed with Gal residue at position 6, specifically the oxygen and one of the two hydrogens with Asn174 and Glu184, respectively. Moreover, OH at position 4 of Gal established a H‐bond with HN of the guanidinium group of Arg162. The Bn moiety was stabilized by van der Waals interactions with the S2–S3 β‐sheets, particularly with Ala146, Asp148, and Ser237. H‐bonds between the oxygen and the hydrogen at position 6 of Gal’ residue was also predicted with the peptide backbone of Trp181 and Asn179, respectively. Bn’ residue was excluded from the Gal‐3 recognition, in agreement with the protein‐based NMR results.

Finally, the role of water molecules in stabilizing the Gal‐3/SeDG‐Bn complex was evaluated during MD simulations. Consistent with previous reports,[^41^, ^50^] conserved water molecules were identified within the binding pocket, mediating critical hydrogen bonds between the ligand and residues such as Arg162, Glu184, and Asn174. The benzyl group of SeDG‐Bn disrupted transient water molecules in proximity to β‐sheets S2 and S3, enhancing hydrophobic contacts with residues Leu147 and Phe149 (Figure S3). These observations underscore the dual role of water molecules in maintaining structural integrity and modulating ligand binding.

## Conclusions

This study provides a detailed exploration of the molecular recognition process between Gal‐3 and the synthetic selenoglycoside‐based ligand SeDG‐Bn.^[35]^ Gal‐3, a multifunctional protein involved in critical biological processes such as cell adhesion, migration, angiogenesis, and carcinogenesis, has become a promising therapeutic target due to its overexpression in several pathological conditions.[^2^, ^8^, ^51^] Specifically, the Gal‐3 CRD plays a key role in recognizing β‐galactose‐containing glycoconjugates, making it a focus for the development of selective inhibitors.[^15^, ^18^, ^19^]

Building on our previous work on SeDG‐Bn inhibitors and their interaction with Gal‐3,^[35]^ this study combines NMR and computational methods to generate a three‐dimensional model of the complex. The introduction of a benzyl group at C3 position of SeDG has proven to significantly enhance the ligand binding affinity toward Gal‐3.^[35]^ STD NMR showed selective recognition of the aromatic benzyl protons, which enhance binding affinity.^[52]^ CSP analysis further pinpointed specific interactions, including hydrogen bonds with Arg162, Asn174, and Glu184, and CH‐π interactions with Trp181, crucial for complex stabilization.^[53]^

MD simulations revealed a mechanism of conformational selection, where the flexibility of SeDG‐Bn in its free state is restricted upon binding to Gal‐3, enhancing binding specificity and stability. Hydration analysis identified conserved water molecules in the Gal‐3 binding pocket stabilizing the ligand‐protein interactions, mediating hydrogen bonds with key residues such as Arg162 and Glu184. The benzyl group also influenced the local hydration environment by displacing transient water molecules, promoting enhanced hydrophobic interactions with residues in β‐sheets S2 and S3. These results are consistent with recent findings also on aralkyl‐thiodigalactosides,^[54]^ which demonstrated that aromatic substituents enhance ligand binding by establishing additional hydrophobic and cation‐π interactions with key residues such as Arg162 and Arg186 in Gal‐3. STD NMR and molecular docking studies of these derivatives further revealed their ability to stabilize the ligand‐protein complex through π‐π stacking interactions with Trp181 and modulation of the hydration environment around the binding pocket. The parallels between these observations and the behavior of the benzyl group in SeDG‐Bn highlight the role of aromatic modifications in increase ligand affinity. Together, these findings describe the dual contribution of both direct ligand‐protein interactions and the surrounding hydration network to the high‐affinity binding of SeDG‐Bn.

Overall, these data provide a detailed understanding of Gal‐3 and SeDG‐Bn 3D complex, demonstrating that the aromatic benzyl group enhances ligand affinity and binding stability compared to the galactose backbone in SeDG, by establishing hydrophobic contacts with key Gal‐3 residues. This observation is consistent with previous studies on other glycomimetics targeting galectin‐3, which have also demonstrated that the introduction of hydrophobic moieties can significantly enhance ligand affinity and binding specificity.[^17^, ^19^, ^55^] The findings highlight the potential of selenium‐containing compounds, such as SeDG‐Bn, as a starting point for the development of novel Gal‐3 inhibitors, which could be further optimized to target diseases like cancer and fibrosis, where Gal‐3 overexpression is implicated.

## Supporting Information Summary

The authors have cited additional references within the Supporting Information.[^56^, ^57^, ^58^, ^59^, ^60^, ^61^, ^62^]

## Conflict of Interests

The authors declare no conflict of interest.

1

## Supporting information

As a service to our authors and readers, this journal provides supporting information supplied by the authors. Such materials are peer reviewed and may be re‐organized for online delivery, but are not copy‐edited or typeset. Technical support issues arising from supporting information (other than missing files) should be addressed to the authors.

Supporting Information

## Data Availability

The data that support the findings of this study are available from the corresponding author upon reasonable request.
