# Supplementary material for: Structural Insights Into Galectin‐3 Recognition of a Selenoglycomimetic
Source: ChemMedChem. 2025 Mar 6;20(10):e202401005. doi: 10.1002/cmdc.202401005 (PMC12091848; doi:10.1002/cmdc.202401005)
Supplement: Supplementary file 1 — Supporting Information [file CMDC-20-e202401005-s001.pdf]

ChemMedChem

Supporting Information

Structural Insights Into Galectin-3 Recognition of a Selenoglycomimetic

Maria Pia Lenza, Cristina Di Carluccio, Ferran Nieto-Fabregat, Luciano Pirone, Rita Russo, Sonia Di Gaetano, Domenica Capasso, Alessia Stornaiuolo, Alfonso Iadonisi, Michele Saviano, Roberta Marchetti, Emilia Pedone,* and Alba Silipo*

SUPPORTING INFORMATION

Structural Insights Into Galectin-3 Recognition Of A Selenoglycomimetic

Maria Pia Lenza,†[a],[b] Cristina Di Carluccio,†b[a],[g] Ferran Nieto-Fabregat,†[a] Luciano Pirone,[c], Rita Russo,[c],[d] Sonia Di Gaetano,[c] Domenica Capasso,[e] Alessia Stornaiuolo, [a] Alfonso Iadonisi,[a] Michele Saviano,[f] Roberta Marchetti,[a] Emilia Pedone,*[c] Alba Silipo*[a],[g]

[a] Dr. M. P. Lenza, Dr. C. Di Carluccio, Dr. F. Nieto-Fabregat, A. Stornaiuolo, Prof. A. Iadonisi, Prof. R. Marchetti, Prof. A. Silipo
Department of Chemical Sciences and Task Force for Microbiome Studies
University of Naples Federico II
Via Cinthia 4, 80126, Naples, Italy.
E-mail: silipo@unina.it

[b] Dr. M. P. Lenza
Department of Pharmacy
University of Naples Federico II
Via Domenico Montesano, 49, 80131 Napoli, Italy

[c] Dr. L. Pirone, Dr. R. Russo, Dr. S. Di Gaetano, Dr. E. Pedone
Institute of Biostructures and Bioimaging
National Research Council (CNR)
Via P. Castellino 111, 80131 Naples, Italy
E-mail: emiliamaria.pedone@cnr.it

[d] Dr. R. Russo
Department of Environmental, Biological and Pharmaceutical Sciences and Technologies
University of Campania "Luigi Vanvitelli"
Via Vivaldi, 43, Caserta, Italy

[e] Dr. D. Capasso
Department of Physics “Ettore Pancini”
University of Naples Federico II
Via Cinthia 4, 80126 Naples, Italy

[f] Dr. M. Saviano
Institute of Crystallography
National Research Council (CNR)
81100, Caserta, Italy

[g] Dr. C. Di Carluccio, Prof. A. Silipo
CEINGE Biotecnologie Avanzate Franco Salvatore
Via Gaetano Salvatore, 486, 80131 Napoli NA

[†] These authors contributed equally

SUPPORTING INFORMATION

Abstract: Chimera-type galectin-3 (Gal-3) is a β-galactoside-binding protein containing a single conserved carbohydrate-recognition domain, crucial in fibrosis and carcinogenesis. Selenium-based Gal-3 inhibitors have emerged as promising therapeutic agents, particularly for treating neoplastic diseases. Among them, a seleno-digalactoside (SeDG) chemically modified by adding a benzyl group to the position 3 of the saccharide residues (SeDG-Bn), attracted a lot of attention for its selectivity and potent inhibitory efficacy against Gal-3. By using a combination of nuclear magnetic resonance and molecular dynamics simulations, we here report the molecular recognition of SeDG-Bn, providing information regarding its epitope mapping and accommodation mode into Gal-3 binding pocket, depicting a 3D model of the complex.

Our findings show that the presence of a single benzyl group establishes hydrophobic contacts with amino acids in the Gal-3's β-sheets S2 and S3, crucially enhancing the binding affinity compared to unmodified SeDG. The Gal-Gal' backbone orientation in the canonical binding site of Gal-3 is partially modified by the benzyl group with respect to Gal-3 complexes with lactosamine and SeDG. These results provide valuable insights into the design of more potent and selective inhibitors for Gal-3, potentially contributing to new therapeutic strategies for conditions such as cancer and fibrosis.

DOI: XXXXXXXXXXXXXX

Table of Contents

Experimental Procedures pag. 3
SeDG-Bn Synthesis pag 3
Protein Preparation pag 3
NMR analysis pag 3
Ligand-based experiments pag 3
STD NMR
CPMG NMR
Protein-based experiments pag 3
Calculation of dissociation constant by NMR Titration pag 4
Molecular Mechanics and Molecular Dynamics simulation pag 4
Supporting Figures pag 5

Experimental Procedures

SeDG-Bn Synthesis.

The SeDG-Bn ligand was synthetized following the previously describe methodology.[35] The αD value was -8.6 (conc. 0.20).

Protein preparation.

The carbohydrate recognition domain of Gal-3 was expressed and purified following the protocol by Pirone *et al*.[34] The isotopically labelled ^{15}N-Gal-3 was expressed in M9 medium by adding biotin, thiamine and ^{15}N ammonium chloride.

NMR Analysis

NMR experiments were performed using a Bruker AVANCE NEO 600 MHz equipped with a cryo probe at 298K. Data acquisition and processing were executed with TOPSPIN 4.1.1 software. Samples were prepared in 50 mM deuterated phosphate buffer (NaCl 140 mM, Na_2HPO_4 10 mM, KCl 3 mM, pH 7.4) and using [D4](trimethylsilyl) propionic acid sodium salt (TPS 0.05 mM) as the internal reference for the spectra calibration.

Ligand-based experiments

STD NMR experiments were acquired with 32 k data points and zero-filled up to 64 k data points prior to processing. Protein resonances were selectively irradiated using 40 Gauss pulses lasting 50 ms, with the off-resonance pulse frequency set at 40 ppm and the on-resonance pulse at 0 ppm, with a relaxation delay D1 of 2 s. A spinlock to suppress protein signals was applied. A mixture of Gal-3 and SeDG-Bn was prepared by using a protein:ligand ratio of 1:65. The %STD values attributed to ligand epitope mapping were derived from the ratio of the STD signals in the STD spectrum (I_0—I_{sat}) and the corresponding peak intensity of the unsaturated reference spectrum (off-resonance, I_0), at saturation time of 2 s. The peak that received the strongest magnetization from the protein (the aromatic protons) was set to 100%, while the STD enhancements of the other protons were normalized to this value. STD NMR experiments at the same conditions were also performed on a solution of the ligand in absence of Gal-3 as control.

CPMG NMR experiments were carried out to determine T2 spin-spin relaxations of the ligand in absence and in presence of the protein. A pseudo 2D sequence with water suppression using excitation sculpting with gradients was considered. A fixed echo time was set to 3 ms and a recycle delay to 2 s. T2 signals were analyzed with Dynamic Center 2.7.1 by fitting the equation f(t) = Io * exp (-t/T).

Protein-based NMR experiments

Uniformly ^{15}N-labeled Gal3 was resuspended in buffer with 20 mM TRIS pH 8, 150 mM NaCl, 1 mM DTT (100 μM) at a final concentration of 100 μM and using [D4](trimethylsilyl) propionic acid sodium salt (TPS 0.05 mM) as the internal reference for the spectra calibration. ^{1}H–^{15}N TROSY HSQC experiments for apo and bound Gal-3 were acquired at 293 K.^{15}N-Gal3 was titrated with increasing equivalents of ligands until complete saturation was achieved. A TROSY experiment was used for which 32 scans were acquired with 256 (t1) × 2048 (t2) complex data points in ^{15}N and ^{1}H, respectively.

CcpNmr Analysis software [56] was employed for data analysis. Average chemical shift changes were calculated by using the following equation[57]

$$\delta_{average} = \sqrt{\frac{1}{2}[\Delta\delta_H^2 + (0{,}14\Delta\delta_N^2)^2]}$$

Calculation of dissociation constant by NMR Titration

The equilibrium dissociation constant (K_d) was determined using NMR titration. Changes in the chemical shift (Δd) were monitored as the ligand concentration increased. K_d was then calculated by numerical fitting to the following equation:

$$\Delta d = \Delta d_{max} \left\{ \frac{([P]_t + [L]_t + K_d) - \sqrt{([P]_t + [L]_t + K_d)^2 - 4[P]_t[L]_t}}{2[P]_t} \right\}$$

In this equation, Δd represents the observed chemical shift difference from the free state, while Δd_{max} is the maximum shift difference observed when the ligand concentration saturates the protein. Both Δd_{max} and K_d were obtained through fitting. $[P]_t$ and $[L]_t$ refer to the total concentrations of protein and ligand, respectively.

Computational Studies

Molecular Mechanics

Molecular mechanics calculations were performed on the Gal1-S-1Gal disaccharide, using the S atom instead of the Se atom. A MM3* force field in the vacuum with a dielectric constant of 80 was used. Both Φ and Ψ were varied incrementally using a grid step of 18°, each (Φ, Ψ) point of the map was optimized using 2000 P.R. The β-Gal residue with the selenium atom in position 1 was built with Gaussian 09 [45] performing the Restrained ElectroStatic Potential (RESP) charges calculation with a Hartree-Fock calculation and a 6-31G* basis set. VFFDT[46]

Docking

Docking calculations were performed using CB-Dock2[47] for cavity detection and blind docking, followed by refined docking with AutoDock Vina.[49] CB-Dock2 was used to identify five potential binding cavities within the Gal-3 carbohydrate recognition domain. Subsequently, AutoDock Vina was employed to dock SeDG-Bn into these cavities. The docking grid was centered around the ligand, with a cubic box of 60 × 60 × 60 Å³ dimensions and an exhaustiveness parameter set to 32 to ensure adequate sampling of binding poses.

The ligand and protein were prepared using AutoDockTools.[58] The ligand was parameterized with all rotatable bonds set as free, and Gasteiger charges were assigned. For the protein, water molecules were removed, and polar hydrogens were added to ensure proper representation of the binding environment.

Docking poses were clustered based on root-mean-square deviation (RMSD) with a cutoff of 2.0 Å, and clusters were ranked according to the binding energy of the lowest-energy representative pose. The structure from the most energetically favorable and most populated cluster was selected as the starting point for molecular dynamics simulations.

Molecular Dynamics simulation

Antechamber and xLeap were combined to generate the .prep and .frcmod files. [59] Molecular dynamic simulations were performed with AMBER 18 software [44] in explicit water. AMBER ff14SB, Glycam06j-1, GAFF2 and TIP3P force fields were used for the protein, the saccharide moiety of the ligands, the aromatic moiety of SeDG-Bn and the water solvent molecules respectively. Additionally, a glycam adapted force field for the Se atom was prepared for the seleoglycosidic linkage. Maestro Protein Preparation Wizard [60] was used to prepare the protein, adding missing hydrogen, the protonation states of ionizable groups and cap termini. The systems were hydrated with an octahedral box containing explicit TIP3P water molecules, buffered at 15 Å. Counterions were added to neutralize the system. Input files were generated using the tleap modules of the AMBER 18 package. For the minimization steps, the Sander module was used, while molecular dynamics calculations were performed with the PMEMD module. An initial energy minimization was conducted to refine the structure, employing SHAKE for C-H bonds with a 1 fs integration step. Periodic boundary conditions and the smooth particle mesh Ewald method, with a grid space of 1 Å, were applied to represent electrostatic interactions. Initially, the system was minimized while holding the complex fixed, followed by a minimization of the entire system. The system was then slowly heated from 0 to 300 K with a weak restraint on the solute. The temperature was increased from 0 K to 100 K at constant volume, and from 100 K to 300 K in an isobaric ensemble. The temperature was maintained at 300 K for 50 ps, with progressive energy minimizations and solute restraint. After equilibration, the system restraints were removed, and the simulations proceeded in an isothermal-isobaric ensemble during the production phase. The system coordinates were saved for the 100 ns simulations using the PMEMD module in AMBER, with coordinate trajectories recorded every 2 ps, resulting in an ensemble of 10,000 structures for each complex, which were subsequently analyzed. Trajectories were analyzed using the ptraj module in AMBER 18, and the VMD program[61] was used to visualize the MD results. Each trajectory underwent cluster analysis based on the ligand RMSD using the K-mean algorithm in the ptraj module. The representative structure of the most populated cluster was used to illustrate the complex interactions.

Hydrogen bond determination was carried out using the CPPTRAJ module in AMBER 18. A hydrogen bond was defined as occurring between an acceptor heavy atom (A), a donor hydrogen atom (H), and a donor heavy atom (D), with a distance cut-off of 3 Å and an A-H-D angle cut-off of 135°. The 3D images were prepared using PyMOL.[62] Dihedral conformation analysis was performed using a custom script to depict torsion variation during the MD simulation and to obtain a histogram of the most populated values.

Supporting Figures

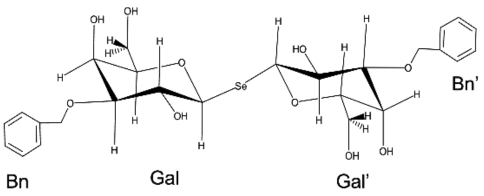

Scheme S1. Chemical structure of SeDG-Bn.

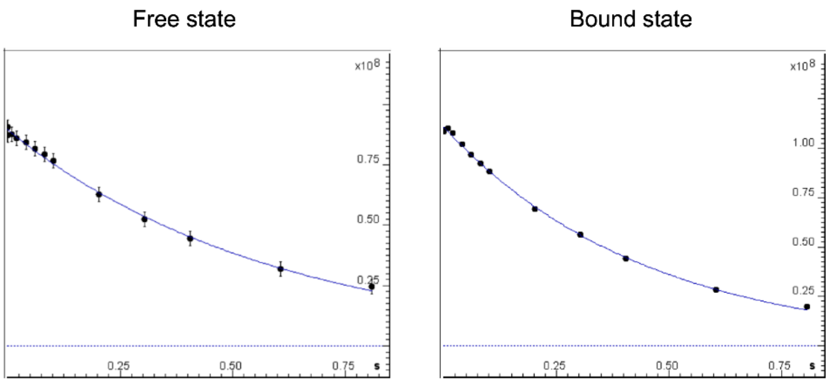

T2 free (s)	Error	T2 bound (s)	Error
0.478	0.032	0.393	0.034

Figure S1. CPMG NMR analysis of SeDG-Bn in free and bound states. An example of T2 decay is reported for proton at position 4 of galactose. T2 values shown in the table were calculated by considering the average of the integrations of different protons' signals.

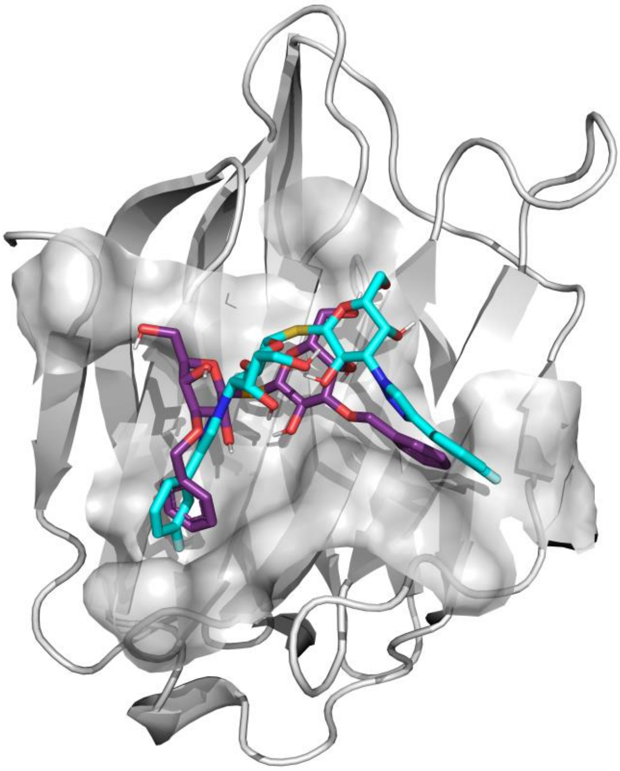

Figure S2. Docking representative pose showing the Gal-3 protein (white) with SeDG-Bn (purple) and TD139 (cyan) from the most populated and less energetic clusters. The surface of amino acid residues within 4 Å of the ligand is highlighted, illustrating key regions of the binding pocket involved in the interaction.

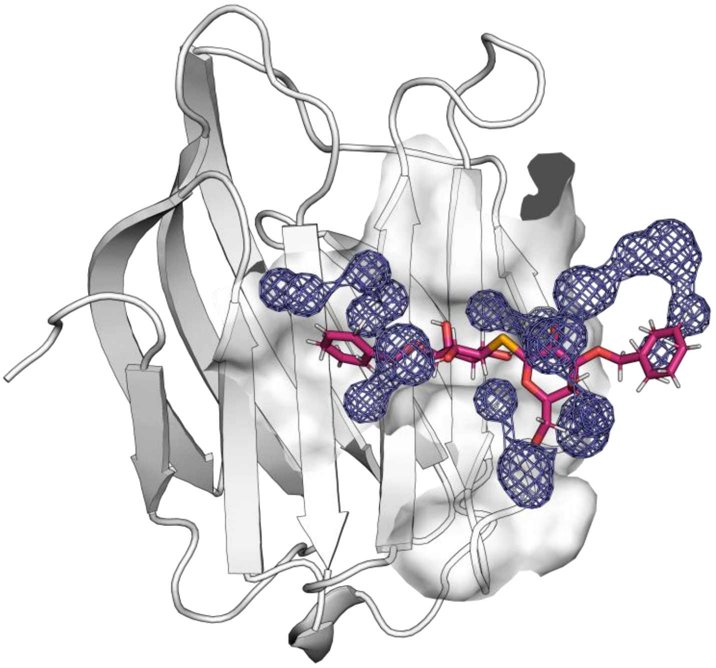

Figure S3. Water density occupancy (blue mesh), calculated over the MD trajectory at a distance of 3 Å around the SeDG-Bn ligand (pink) and the binding pocket residues. The Gal-3 protein is represented in white, highlighting the hydration environment critical for ligand stabilization.
